# Distribution and utilization of vector control strategies in a malarious village of Jabi Tehnan District, north-western Ethiopia

**DOI:** 10.1186/1475-2875-13-356

**Published:** 2014-09-10

**Authors:** Abebe Animut, Yohannes Negash, Nigatu Kebede

**Affiliations:** Aklilu Lemma Institute of Pathobiology, Addis Ababa University, P O Box 1176, Addis Ababa, Ethiopia

## Abstract

**Background:**

Adequate coverage and proper use of long-lasting insecticidal nets (LLINs) and indoor residual spaying (IRS) reduce density of indoor-resting mosquitoes, man-mosquito contact and malaria infection. However, distribution, ownership and usage of the interventions may vary among households in a malarious area, which in turn limits the impact of interventions on the transmission of malaria. A study was undertaken to assess distribution and utilization of LLINs and IRS in a malarious village of north-western Ethiopia.

**Methods:**

A total of 352 randomly selected households in Jiga Yelmdar village, Jabi Tehnan District of north-western Ethiopia were interviewed using a structured questionnaire. The most important questions included distribution and utilization of LLINs/IRS and knowledge by the households of malaria and the interventions.

**Results:**

More than 99% of the respondents had information about malaria. About 97% of the households had at least one LLIN and 89.3% of houses had been treated with IRS within the previous six months. Only 58.2% of the LLIN-owning households had used the nets the previous night. Not being a malaria transmission season was the main reason cited by 69.7% of the households for not using their LLINs the previous night. The most preferred malaria control strategy in the village was LLINs (cited by 71.1%) followed by IRS (cited by 14.5%). About 29% of the households had a history of malaria within the previous six months and the great majority of them (86.3%) sought treatment at the Jiga Yelmdar Health Post or Jiga Health Centre.

**Conclusion:**

Residents of Jiga Yelmdar village were knowledgeable about malaria and the control strategies of the disease such as LLINs and IRS. Although LLIN use is their most preferred strategy, the compliance rate was low which probably contributed to the 29% of household-level malaria infection in the village within the previous six months. This indicates the need for improved compliance to LLINs and IRS in the village.

## Background

Long-lasting insecticidal nets (LLINs) and indoor residual spaying (IRS) are the vector control strategies that have contributed to the current reduction in the global malaria burden [1, 2]. LLINs are used for personal protection against nuisance biting insects, including malaria vectors. They also protect individuals who reside within a few metres of LLIN-using households [3]. IRS saved lives in Europe, Asia and the Americas between the 1940s and the 1980s [2]. It rapidly reduced density and longevity of anopheline mosquitoes and therefore malaria transmission, when properly applied [1, 2]. IRS can also be combined with LLINs in areas where vectors develop resistance to the insecticides used to impregnate net fabrics [2].

Adequate coverage [4, 5] and proper use of LLINs and IRS reduce the density of indoor-resting mosquitoes, man-mosquito contact and malaria infection [4, 6–9]. The Federal Ministry of Health (FMoH) of Ethiopia has been undertaking a scaled-up distribution of LLINs and IRS as its major malaria control strategies in malarious areas of the country, depending on the local epidemiology of the disease [10]. However, distribution, ownership and usage of the interventions may vary among households and may limit the impact of the interventions on overall malaria transmission in the area. In addition, households need to have knowledge of the strategies and their benefits if proper implementation is sought [2, 11, 12]. A study in 17 malarious districts of the country showed distribution and utilization of LLINS to be 97.6 and 81.6%, respectively [13]. In addition, in Arbamich Town and its surrounding district, net coverage and utilization was observed to be 75.1 and 71%, respectively [14]. Other studies also support this trend [7, 15]. Coverage and utilization of interventions should be assessed and findings communicated to health planners for timely corrective measures and early preparedness. In addition, information on the knowledge and perception by rural households in north-western Ethiopia is limited. This study investigated coverage and utilization of LLINS and IRS and knowledge by inhabitants of these strategies in a malaria-endemic district of north-western Ethiopia.

## Methods

### Study area and design

Jabi Tehnan is one of the malaria-endemic districts (district refers to *Woreda* in the Ethiopian context), in the West Gojjam Zone, north-western Ethiopia. The district is bordered on the southeast by Dembecha, on the west by Bure, on the north-west by Sekela, on the north by Quarit and on the east by Dega Damot districts. These bordering districts are malarious.

The district encompasses 41 villages (village refers to *Kebele* in the Ethiopian context), which are malarious. Among these, Jiga Yelmdar was selected randomly (using lottery method). The village had one health post (Jiga Yelmdar Health Post) and one health centre (Jiga Health Centre). It had 962 households during the study period. LLIN distribution and IRS have been undertaken by the district’s health bureau on a regular basis. New LLIN distribution was made in July and August, 2013. In addition, IRS was undertaken in the village in September, 2013 (pers communication, health workers of the village and Jabi Tehnan District). Anticipating the proportion of 50% of household heads who had heard of both LLINs and IRS, with 95% confidence interval and a 0.05 absolute precision, a sample size of 384 households was considered in the study.

### Data collection

The 384 household samples were randomly distributed to a total 962 households in the village, first by interviewing a household head in the northern extreme of the village and then by interviewing the next (962/384)^th^ or third household to the southern direction. In case of absence or refusal by the selected household, the next household was considered in the sample. Data were collected in January 2014 using a pre-tested questionnaire developed in English and then translated into the local language (Amharic). The household head or the next elder of each selected household was interviewed. The major variables included in the questionnaire were socio-economic characteristics of households, knowledge of households about malaria interventions, availability of LLINs in households, number of nets used in households, condition of nets, net utilization, history of IRS, history of wall re-plastering and use of LLINs and IRS. Net condition was further divided into good (clean and having no hole), fair (not clean and having no hole), poor (having at least one hole of about 2 cm in diameter) and still in the package.

### Data analysis

Data were entered and analysed using PASW 18.0 Statistics Version 18 (SPSS Inc, Chicago, IL, USA). The frequency of respondents against the target variable was determined. Association of the variables with the history of malaria infection within the previous six months was analysed using logistic regression.

### Ethical issues

The study was reviewed and approved by the Institutional Review Board of the Aklilu Lemma Institute of Pathobiology, Addis Ababa University. Permission to undertake the study was obtained from West Gojjam Zone and Jabi Tehnan district health bureaus. Informed verbal consent was obtained from all the respondents of the selected study households after the study was explained to them in the local language.

## Results

The socio-demographic characteristic of the surveyed households is presented in Table 1. A total of 352 households were visited and female respondents (226; 64.2%) were 1.8 times higher than the males (126: 35.8%). The mean age of the respondents was 36.4 years old ranging from 18 to 80 years. The highest proportion of respondents were illiterate (47.7%) followed by able to read and write (22.2%), primary school complete (16.2%), secondary school (8.2%) and high school or higher (5.7%). The great majority of the respondents were farmers (93.8%). About 53% of them did not own any type of media while 46.3% had radio. The average household size of the village was 4.81 persons.

**Table 1:** **Socio-demographic characteristics of households in Jiga Yelmdar village, Jabi Tehnan District, north-western Ethiopia, January 2014**

Characteristic	Category	Number (%)
Sex of respondents (n = 352)		
	Male	126 (35.8)
	Female	226 (64.2)
Educational status of respondents (n = 352)	Illiterate	168 (47.7)
	Read and write	78 (22.2)
	Primary school	57 (16.2)
	Secondary school	29 (8.2)
	High school or higher	20 (5.7)
Household occupation (n = 352)	Farmer	330 (93.8)
	Merchant	8 (2.3)
	Government employee	2 (0.6)
	Other	12 (3.4)
Media ownership of households (n = 352)	Radio	163 (46.3)
	Television	1 (0.3)
	Newspaper	2 (0.6)
	No	186 (52.8)
Household size (n = 352)	1	6 (1.7)
	2	26 (7.4)
	3	65 (18.5)
	4	60 (17.0)
	5	73 (20.7)
	6	55 (15.6)
	7	35 (9.9)
	8	28 (8.0)
	≥9	4 (1.2)

More than 99% (n = 350) of the respondents had information about malaria and the most frequent information source cited was health workers (83.2%) (Table 2). The majority (89.5%) of respondents reported that they use LLINs to prevent malaria. Almost all (98.9%) of the respondents had heard about LLINs and most (96.9%) of them owned at least one LLIN. The households that owned one, two, three, and four or higher LLINs were 136 (39.9%), 182 (53.4%), 18 (5.3%), and 5 (1.5%), respectively.

**Table 2:** **Knowledge by households of malaria and long-lasting insecticide nets in Jiga Yelmdar village, Jabi Tehnan District, north-western Ethiopia, January 2014**

Characteristic	Category	Number (%)
Heard about malaria (n = 352)	Yes	350 (99.4)
	No	2 (0.6)
Information source about malaria (n = 351)	Health worker	292 (83.2)
	Mass media	21 (6.0)
	Friend	23 (6.6)
	Other	15 (4.3)
Method used to prevent malaria (n = 352)	LLINs	315 (89.5)
	IRS	11 (3.1)
	Aerosol spray	1 (0.3)
	Anti-malarial tablet	10 (2.8)
	Clean surroundings	10 (2.8)
	Habitat management	2 (0.6)
	Other	3 (0.9)
Heard about LLINs (n = 352)	Yes	348 (98.9)
	No	4 (1.1)
Have LLINs (n = 352)	Yes	341 (96.9)
	No	11 (3.1)
Number of LLINs per household (n = 341)	1	136 (39.9)
	2	182 (53.4)
	3	18 (5.3)
	≥4	5 (1.5)

About 93% (328/352) of the respondents perceived that LLINs are used to prevent malaria (Table 3). While 6.3% (n = 22), 0.3% (n = 1) and 0.3% (n = 1) cited that LLINs are used to prevent mosquitoes, insects and rodents, respectively. About 81% (276/341) of the respondents reported current LLIN use in their households of whom 58.2% (198/340) had slept under an LLIN the previous night. LLIN use by all the family was cited most (57.6%; 197/342) followed by use by father and mother (17.8%; 61/342) and use by children and mother (10.2; 35/342). The most cited reason for not sleeping under the available LLIN was that it was not the malaria season (69.7%; 99/142) followed by others (29.6%; 42/142). Most of the LLINs surveyed (86.5%, 204/342) were in a good condition, while the remainder were poor (7.6%; 26/342), fair (4.7%; 16/342) and unused (1.2%; 4/342). Most (96.3%) of the household respondents have been using LLINs for over three years.

**Table 3:** **Households’ knowledge and utilization of long-lasting insecticide nets by households in Jiga Yelmdar village, Jabi Tehnan District, north-western Ethiopia, January 2014**

Characteristic	Category	Number (%)
Use of LLINs (n = 352)	Prevent malaria	328 (93.2)
	Kill/prevent mosquito	22 (6.3)
	Kill/prevent insects	1 (0.3)
	Kill/prevent rodent	1 (0.3)
Current LLINs used (n = 341)	Yes	276 (80.9)
	No	65 (19.1)
Previous night LLINs used (n = 340)	Yes	198 (58.2)
	No	142 (41.8)
Reason for not using LLINs (n = 142)	LLINs are toxic	1 (0.7)
	Not malaria season	99 (69.7)
	Other	42 (29.6)
Who uses LLINs (n = 342)	Children	16 (4.7)
	Mother	13 (3.8)
	Father	20 (5.8)
	Father and mother	61 (17.8)
	Children and mother	35 (10.2)
	Whole family	197 (57.6)
Frequency of LLIN use (n = 342)	Daily	204 (59.6)
	Occasionally	138 (40.4
Condition of LLINs (n = 342)	Good	296 (86.5)
	Fair	16 (4.7)
	Poor	26 (7.6)
	Unused (still in package)	4 (1.2)
Period since LLIN used (n = 348)	<1 month	1 (0.3)
	Months ago	8 (2.3)
	1-3 years	4 (1.1)
	>3 years	335 (96.3)

Almost all (99.4%; 350/352) of the respondents have heard about IRS of which the majority (63.4%; 223/352) did not know the insecticide type employed (Table 4). A high proportion (67%; 236/252) of the total respondents perceived that IRS is used to kill mosquitoes. About 89.3% (275/308) of the houses had been treated with IRS within the previous six months and most of them had not been plastered since the last treatment with insecticide.

**Table 4:** **Coverage and households’ perception of indoor residual spraying in Jiga Yelmdar village, Jabi Tehnan District, north-western Ethiopia, January 2014**

Characteristic	Category	Number (%)
Heard about IRS (n = 352)	Yes	350 (99.4)
	No	2 (0.6)
Insecticide employed for IRS (n = 352)	DDT	125 (35.5)
	Malathion	1 (0.3)
	Deltamethrin	1 (0.3)
	Other	2 (0.6)
	Do not know	223 (63.4)
Part of the house for IRS (n = 352)	Surface of inner wall	338 (96.0)
	Inner surface of the roof	4 (1.1)
	All parts	8 (8)
	Do not know	2 (0.6)
Use of IRS (n = 352)	To kill mosquitoes	236 (67.0)
	To kill other domestic insects	38 (10.8)
	To kill rodents	1(0.3)
	To control malaria	75 (21.3)
	Do not know	2 (0.6)
House treated by IRS within past 12 months	Yes	308 (87.5)
	No	44 (12.5)
When was the last IRS (n = 308)	>3 months	275 (89.3)
	>6 months	34 (10.7)
Frequency of IRS (n = 322)	Once per year	210 (65.2)
	After every six months	105 (32.6)
	After every three months	4 (1.2)
	Other	1 (0.3)
	Do not know	2 (0.6)
Time of IRS	In the morning (06:00-12:00)	91 (28.3)
	At mid day (12:00-13:00)	114 (35.4)
	Afternoon (13:00-18:00)	104 (32.3)
	Any time (06:00am-18:00)	13 (4.0)
Wall plastered since last IRS (n = 316)	Yes	29 (9.4)
	No	279 (90.6)

The most preferred malaria prevention strategy in Jiga Yelmdar was LLINs (74.1%; 261/352) followed by IRS (14.5%; 51/352), larval source management (6.3%; 22/352) and case treatment (2.6%; 9/352) (Table 5). About 29% (n = 102) of the respondents perceived a history of malaria infection in their households within the previous six months. The majority of infected household members (86.3%; 88/102) sought treatment in health posts/centres followed by the cases who bought drugs from drug stores (6.9%; 7/102).

**Table 5:** **Reported malaria infection among households within the previous six months and preferred control/treatment method, in Jiga Yelmdar village, Jabi Tehnan District, north-western Ethiopia, January 2014**

Characteristic	Category	Number (%)
Preferred malaria prevention method for the household (n = 352)	LLINs	261 (74.1)
	IRS	51 (14.5)
	Case treatment	9 (2.6)
	Larval source management	22 (6.3)
	Do not know	9 (2.6)
Malaria infection history in household within previous 6 months (n = 352)	Yes	102 (29.0)
	No	250 (71.0)
Treatment sought (n = 102)	Health post/centre	88 (86.3)
	Hospital	1 (1.0)
	Traditional healer(s)	3 (2.9)
	Drug store (pharmacy)	7 (6.9)
	Nowhere	3 (2.9)

## Discussion

Almost all of the household respondents had information about malaria, LLINs and IRS and they cited health workers as their main source of information. The village health extension workers undertake a regular house-to-house education on disease control and prevention methods, including malaria. They diagnose febrile patients for malaria clinically and using rapid diagnosis test kits, and treat positive cases following national diagnosis and treatment guideline [10]. Diagnosis and treatment of malaria cases is carried out free of charge at the Jiga Yelmdar Health Post. The house-to-house education and the free treatment of cases at the health post might have contributed to increased awareness and treatment-seeking behaviour of households, respectively. This is central for effective implementation of LLINs and IRS in rural malarious areas.

A great majority of the respondents indicated that LLINs prevent malaria. LLINs can reduce malaria transmission especially among children in endemic areas aged under five years [3, 9, 12, 16]. This results from the killing, repellency and physical protective effects of the LLINs against mosquitoes [3, 17]. The mosquito-killing effect of LLINs reduces the age, human contact and sporozoite rate of anophelines. This reduces malaria infection risk among nearby households having no LLINs and IRS [4]. The nets also serve as physical barriers against nuisance biting mosquitoes and insects, irrespective of resistance [3]. Deltamethrin-treated LLINs were observed to have a 60% malaria preventive effect in an area of highly resistant vectors to the insecticide [9].

In the village, 56.9% (n = 198) of the households owning LLINs and 58.2% (n = 198) of households reporting current net use perceived use of the nets the previous night. This compliance rate is lower compared with the report from Oromia and Amhara Regions (65%) [18], the Southern Nations, Nationalities and People’s Region (SNNPR) (60.6%) [15] and the 17 malarious districts (81.6%) [13] of the country. In this cross-sectional study, which was undertaken during a dry season, the most frequently cited reason for not sleeping under an LLIN the previous night (by 69.7% of the 142 non-compliants) was that it was not a malaria season. This is different from the study in the SNNPR where the main reason mentioned was that nets were too torn [15]. Although the households in Jiga Yelmdar village could experience a lower density of night-biting mosquitoes and possibly lower number of malaria cases during the study period (which was dry) compared with the wet season, this could contribute to a sustained transmission of the disease in the area. The Jiga Yelmdar Health Post reports and entomological collections undertaken in the village revealed occurrence of malaria transmission during the study season (AA, unpublished data).

A high proportion of the respondents (67%; 236/252) perceived that IRS is used to kill mosquitoes. This indicates that the residents are knowledgeable about the use of IRS. This proportion is higher than that reported from Uganda (48.6%) [8] although the areas are different. However, more than half of the respondents did not know name of the chemical used for the IRS. This indicates the need to inform inhabitants about the nature of the chemicals being used for IRS, including names, uses and possible side-effects on animals and humans.

Despite the scaled-up coverage of LLINs and IRS in the Jiga Yehmdar village, a high proportion of respondents (29%) had had malaria infection within the previous six months. This could result from the low level of compliance to LLINs and possibly from resistance of the vectors to the insecticides impregnated in the nets (deltamethrin, perimethrin) and also to chemicals used for IRS (dichlorodiphenyltrichloroethane, malathion) [19–21]. Improved compliance to LLINs can bring a significant reduction in malaria infection risk as the nets provide physical protection even in the presence of insecticide resistance [9]. However, the status of behavioural resistance of the vectors to the LLINs and IRS that are being used in the area, the biting behaviour of vectors before bedtime and their possible outdoor-feeding behaviour remains to be described in the area. This is because the use of LLINs/IRS may force vectors to change their endophilic and endophagic behaviour into exophilic and exophagic [22] and also their biting hours from the period during human sleeping to before bed time [17, 23].

## Conclusion

Residents of Jiga Yelmdar village, Jabi Tehnan District, north-western Ethiopia were aware of malaria and the currently employed control strategies, LLINs and IRS, against the disease. Although LLINs were distributed to almost all households and was the most preferred malaria control strategy in the village, the compliance rate (58.2%) was low which probably contributed to the 29% household-level reported malaria infection. This study indicates the importance of describing seasonal dynamics and behaviour of local vectors. In addition, compliance of households to malaria interventions (LLINs and IRS) needs to be improved through education and regular supervision.
